# Reduced hematopoietic-inflammatory response and worse outcomes in patients with recurrent myocardial infarction in comparison with primary myocardial infarction

**DOI:** 10.1186/s13550-023-01035-9

**Published:** 2023-09-25

**Authors:** Yao Lu, Jingjing Meng, Mingkai Yun, Marcus Hacker, Xiang Li, Xiaoli Zhang

**Affiliations:** 1 Department of Nuclear Medicine, Molecular Imaging Lab, Beijing Anzhen Hospital, Capital Medical University, Beijing, China; 2 Division of Nuclear Medicine, Department of Biomedical Imaging and Image-Guided Therapy, Vienna General Hospital, Medical University of Vienna, Vienna, Austria

**Keywords:** Recurrent myocardial infarction, Bone marrow, Hematopoiesis, Positron emission tomography

## Abstract

**Background:**

Recurrent myocardial infarction (RMI) portends an unfavorable outcome, which might be related to diminished hematopoietic-inflammatory activation. We aimed to investigate the hematopoietic-inflammatory activation and the outcome in categorized patients with primary myocardial infarction (PMI) versus RMI as well as chronic stable angina (CSA) by ^18^F-FDG PET.

**Results:**

A total of 105 patients (88 males; 60.1 ± 9.7 years) were included. Target-to-background ratio of bone marrow (TBR_BM_) was highest in the PMI group (n = 45), intermediate in the RMI group (n = 30), and lowest in the CSA group (n = 30) (P < 0.001). RMI group exhibited larger scar, significantly reduced left ventricular ejection fraction, and enlarged end systolic volume in comparison with the PMI and CSA groups, respectively (P < 0.05). Additionally, there was a significantly positive correlation between TBR_BM_ and TBR_aorta_ (P < 0.001). The cumulative major adverse cardiac events free survival of patients in the RMI group was lower than that in the PMI and CSA groups during a median follow-up of 16.6 months (P = 0.026).

**Conclusions:**

RMI conferred relatively decreased hematopoietic-inflammatory activation compared with PMI. Patients with RMI presented subsequent enlarged myocardial scar, worsened cardiac dysfunction, aggravated remodeling, and worse outcomes than that in PMI patients.

## Introduction

Recurrent myocardial infarction (RMI) after an acute coronary syndrome is associated with poor outcome and high morbidity and mortality [1]. Although RMI rates have declined over time, mortality rates remain high. RMI has a 1-year mortality rate of about 25% based on data from a large real-world cohort in China [2]. The poor outcome after RMI is attributed to additional loss of viable myocardium and heart failure. However, the pathophysiological basis for RMI remains unclear.

Newly-made leukocytes enter the heart and deploy a variety of molecular mediators to enable myocardial healing in myocardial infarction (MI) patients. A sufficient inflammatory reaction is necessary for myocardial healing after MI, while unrestrained myeloid cell activity results in adverse cardiac remodeling and heart failure [3]. The immune response to MI is the massive recruitment of neutrophils and monocytes from hematopoietic organs, which increase myeloid cell production by a process called emergency hematopoiesis [4]. The proliferation of monocyte progenitors and proinflammatory activation of monocytes within the hematopoietic tissues may play an important role in accelerating atherosclerosis after acute MI [5, 6].

Cremer et al. have found that RMI was associated with a state of immune tolerance characterized by reduced emergency hematopoiesis and leukocytosis in the ischemic heart compared with primary MI (PMI) [7]. This mechanism initiated by bone marrow (BM) and spleen has been studied in RMI mouse models but not in humans. It is unclear whether RMI elicits a response identical to that observed after a PMI in humans.

^18^F-fluorodeoxyglucose (^18^F-FDG) could be up-taken by activated inflammatory cells that accumulate in the site of lymphoid organs [8, 9]. Additionally, cellular accumulation of ^18^F-FDG is increased in rapidly proliferating cells. The metabolic activity of the hematopoietic organs (BM and spleen) can also be non-invasively evaluated by ^18^F-FDG PET with the measurement of proliferative activity [10].

In this pilot study, we aimed to investigate the hematopoietic-inflammatory activation and the outcome in categorized patients with PMI versus RMI as well as chronic stable angina (CSA) by ^18^F-FDG PET.

## Methods

### Study population

We retrospectively enrolled patients who underwent gated ^99m^Tc-sestamibi myocardial perfusion imaging (MPI) and ^18^F-FDG cardiac PET as for myocardial viability assessment to guide the treatment strategy decision making at the department of nuclear medicine, Beijing Anzhen Hospital, affiliated with Capital Medical University from January 2019 to January 2021, and diagnosed with PMI, RMI, or CSA, respectively. Patients with MI (PMI and RMI) were examined at 30.0 (interquartile range: 25.0–40.0) days. The study protocol was approved by the ethics committee of Beijing Anzhen Hospital, affiliated with Capital Medical University (Approval No. 2017024).

PMI was defined as typical changes in biochemical markers of myocardial necrosis along with ≥ 1 of the following: ischemic symptoms, electrocardiographic changes indicative of new ischemia, development of pathological Q waves, or imaging evidence of new loss of viable myocardium or new regional wall motion abnormalities [11]. For inclusion, patients with RMI were required to have received at least two distinct diagnoses of MI within a 6-month period, defined as elevated cardiac enzymes above the diagnostic threshold, with positive high-sensitive troponin-I (hsTnI) values recorded at least once within 96h after MI [7], along with a typical clinical presentation or typical electrocardiographic changes. CSA was defined as the presence of stable anginal symptoms for ≥ 6 months with ≥ 50% luminal narrowing in ≥ 1 major coronary artery on angiography. Patients with a history of an inflammatory condition, or those taking inflammation-modulating medications within the prior 6 months, malignancy, or severe hepatic disease were excluded.

The following clinical characteristics were recorded: age, sex, body mass index, history of hypertension, diabetes mellitus, dyslipidemia, and smoking. Cardiac hsTnI, creatine kinase-MB and brain natriuretic peptide (BNP) fractions were measured.

### MPI

As previously reported [12], MPI was performed 90–120 min after injection of ^99m^Tc-sestamibi (740 MBq, Chinese Atomic Energy Institute, Beijing, China). Images were acquired for 10 min with a dual-headed Siemens Camera (Siemens Symbia Intevo 16 Systems), equipped with a multifocal (SMART ZOOM) collimator. Gated data were acquired with a 20% energy window centered over 140 keV. Images were reconstructed with the flash 3D mode and displayed as short axis and horizontal, and vertical long-axis slices.

### ^18^F-FDG cardiac PET imaging

As previously described, patient preparation followed the protocol as outlined in the 2016 American Society of Nuclear Cardiology (ASNC) guidelines [13]. After at least 12 h of fasting, the blood glucose level was controlled by oral glucose loading and, if needed, by supplemental iv insulin doses as recommended in the ASNC guidelines [13]. Blood glucose levels averaged 5.88 ± 0.67 mmol/L at the time of the injection of ^18^F-FDG (Chinese Atomic Energy Institute, Beijing, China). ^18^F-FDG cardiac PET was acquired for 10 min. Attenuation correction was performed based on CT data (120 kV, 11 mAs). Image reconstruction employed a point spread function + time of flight (TOF) algorithm (TrueX + TOF, UltraHD-PET), with 2 iterations and 21 subsets (Siemens AG, Munich, Germany).

### Imaging analysis

^18^F-FDG metabolic activity in BM was calculated under CT-guided anatomic reference from the fifth to eighth thoracic vertebrae according to our previous investigation [14]. Besides, the average of the highest standard uptake value (SUV) was used as the mean SUV, and normalized to the right atrium. Splenic ^18^F-FDG metabolic activity was assessed by placing regions of interest in 3 planes (axial, sagittal, and coronal planes), SUVmax was recorded in each plane, and the splenic activity was calculated as the mean of SUVmax values of the 3 planes and normalized to the liver. Aortic ^18^F-FDG metabolic activity was quantified in the region of interest around each aorta on every slice of the trans-axial fusion PET/CT images. The highest SUV of the region of interest of all 3 slices within the aorta was averaged for each subject [15]. Thus, the aorta SUV was divided by the blood-pool SUV measured from the right atrium for normalization. In this way, the aortic target-to-background ratio (TBR) was calculated for each subject.

Left ventricular (LV) global functional and remodeling parameters, including LV ejection fraction (LVEF, %), end-diastolic volume (EDV, mL), and end-systolic volume (ESV, mL) were calculated by using QGS software (version 3.1, Cedars-Sinai Medical Center, Los Angeles, CA, USA), with manual correction in case of the inadequate endocardium and epicardium delineation [16]. As in our previous investigations [17, 18], myocardial perfusion and metabolic activity were assessed by two experienced physicians using the American Heart Association 17-segment and five-point scoring system. Hibernating myocardium (HM, %) was defined as a mismatch score of 1.0 or greater (perfusion score minus metabolism score ≥ 1). The infarcted tissue (scar, %) was defined as a mismatch score of less than 1.0 (perfusion score minus metabolism score < 1). One segment accounted for 6% of LV; the extent of HM and scar were calculated from the number of segments with mismatches or matches while the total perfusion deficit (TPD, %) was derived from the number of hypo-perfused segments and their deficit severity [19].

### Follow-up

Follow-up was performed by consulting the electronic medical record system and contacting patients or their relatives by telephone. The primary endpoint was major adverse cardiac events (MACE), including all-cause death, cardiac death, MI, and readmission due to heart failure. The median follow-up time was 16.6 months (interquartile range 11.7–32.0 months).

### Statistical analysis

The baseline characteristics of the patients were analyzed according to the 3 groups. Frequencies and proportions were reported for categorical variables, and either mean ± SD or median with interquartile range was reported for continuous variables based on normality of distribution. The χ^2^ test, Fisher exact test, 1-way ANOVA, and Kruskal Wallis test were used to compare variables among groups. Subsequent comparisons were performed by the Bonferroni post hoc test, Mann–Whitney U test, or Fisher exact test with Bonferroni-corrected P values. The ^18^F-FDG metabolic activity of the BM and spleen aorta were compared among the 3 groups using ANOVA with Bonferroni for multiple comparisons. Spearman correlation analysis was performed to identify the relationship between ^18^F-FDG metabolic activity in BM, spleen, and aorta. The cumulative incidence of MACE-free was estimated by the Kaplan–Meier curve and compared by the log-rank test. Data were analyzed using SPSS for 26.0 (SPSS, Chicago, IL). Statistical significance was defined as P < 0.05.

## Results

### Clinical and laboratory characteristics

A total of 105 patients (88 males; 60.1 ± 9.7 years) were included. Forty-five patients were in the PMI group, and thirty patients were in the RMI and CSA groups, respectively. Nevertheless, the prevalence of traditional cardiovascular risk factors, such as hypertension, diabetes mellitus, dyslipidemia, and smoking were not significantly different among the three groups. Meanwhile, the level of C-reactive protein, white blood cells, neutrophils, high-sensitive Troponin I and BNP did not differ significantly among the three groups. The baseline characteristics of patients are summarized in Table 1.

**Table 1:** Baseline characteristics of study population

	**PMI (n=45)**	**RMI (n=30)**	**CSA (n=30)**		**p value**	
Age, y	61.0 (15.0)	62.5 (18.0)	63.0 (8.0)		0.800	
Men, n (%)	38 (84.4)	26 (86.7)	24 (80.0)		0.841	
BMI, kg/m^2^	25.2 ± 3.8	25.3 ± 3.3	25.9 ± 3.0		0.645	
Hypertension, n (%)	23 (53.5)	17 (56.7)	21 (70.0)		0.344	
Diabetes, n (%)	16 (37.2)	17 (56.7)	16 (53.3)		0.223	
Dyslipidemia, n (%)	19 (44.2)	11 (36.7)	11 (36.7)		0.788	
Smoking, n (%)	17 (37.8)	19 (63.3)	11 (36.7)		0.061	
Total cholesterol, mg/dL	3.92 (1.66)	3.77 (0.71)	3.87 (1.25)		0.664	
Triglycerides, mg/dL	1.37 (0.58)	1.77 (0.85)	1.49 (0.66)		0.129	
HDL-C, mg/dL	0.93 (0.32)	0.93 (0.30)	0.89 (0.25)		0.634	
LDL-C, mg/dL	2.30 (1.06)	2.14 (0.91)	2.23 (0.98)		0.255	
WBC, × 10^9^/μL	8.41 (3.93)	8.39 (4.14)	7.34 (1.85)		0.145	
Neutrophil	6.50 (5.12)	5.33 (3.39)	4.92 (1.26)		0.100	
CRP, mg/L	3.40 (4.68)	2.54 (4.43)	1.81 (2.37)		0.118	
hsTnI, pg/mL	28.5 (105.0)	32.7 (41.0)	11.9 (34.0)		0.152	
CK-MB,ng/mL	1.90 (3.88)	2.10 (1.85)	1.80 (1.78)		0.336	
BNP, pg/mL	311.0 (248.0)	332.0 (328.0)	236.0 (205.0)		0.234	
Revascularization	33 (73.3)	22 (73.3)	21 (70.0)		0.961	

BMI = body-mass index; BNP = Brain natriuretic peptide; CK-MB = creatine kinase-MB; CSA = chronic stable angina; CRP = C-reactive protein; HDL-C = high-density lipoprotein cholesterol; hsTnI = high-sensitive Troponin I; LDL-C = low-density lipoprotein cholesterol; PMI, primary myocardial infarction; RMI, recurrent myocardial infarction; WBC, white blood cell

### Comparison and correlations of ^18^F-FDG metabolic activity in the BM, spleen, and aorta among the three groups

**Fig. 1:**
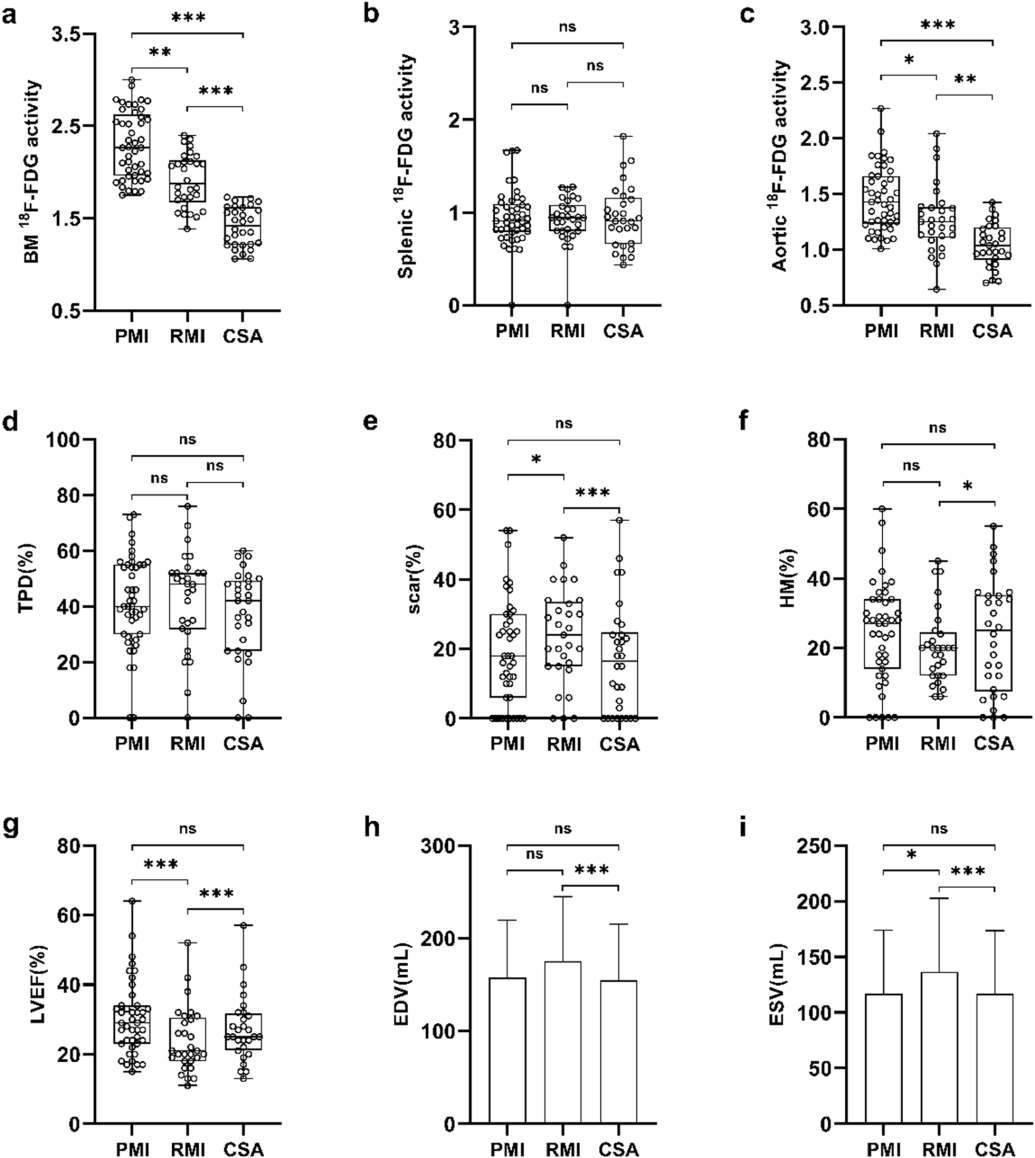
BM, Splenic, and aortic ^18^F-FDG activity among the three groups. The ^18^F-FDG activity of BM (**a**) and aorta (**c**) was highest in the PMI group, intermediate in the RMI group, and lowest in the CSA group. The splenic (**b**) ^18^F-FDG activity did not differ significantly among the three groups. TPD (%, **d**), Scar (%, **e**), HM (%, **f**), LVEF (%, **g**), EDV (mL, **h**), and ESV (mL, **i**) among the three groups. BM, bone marrow; CSA, chronic stable angina; EDV, end-diastolic volume; ESV, end-systolic volume; HM, hibernating myocardium; LVEF, left ventricular ejection fraction; PMI, primary myocardial infarction; RMI, recurrent myocardial infarction; TPD, total perfusion deficit. *P < 0.05. **P < 0.01. ***P < 0.005. ns, no significance

**Fig. 2:**
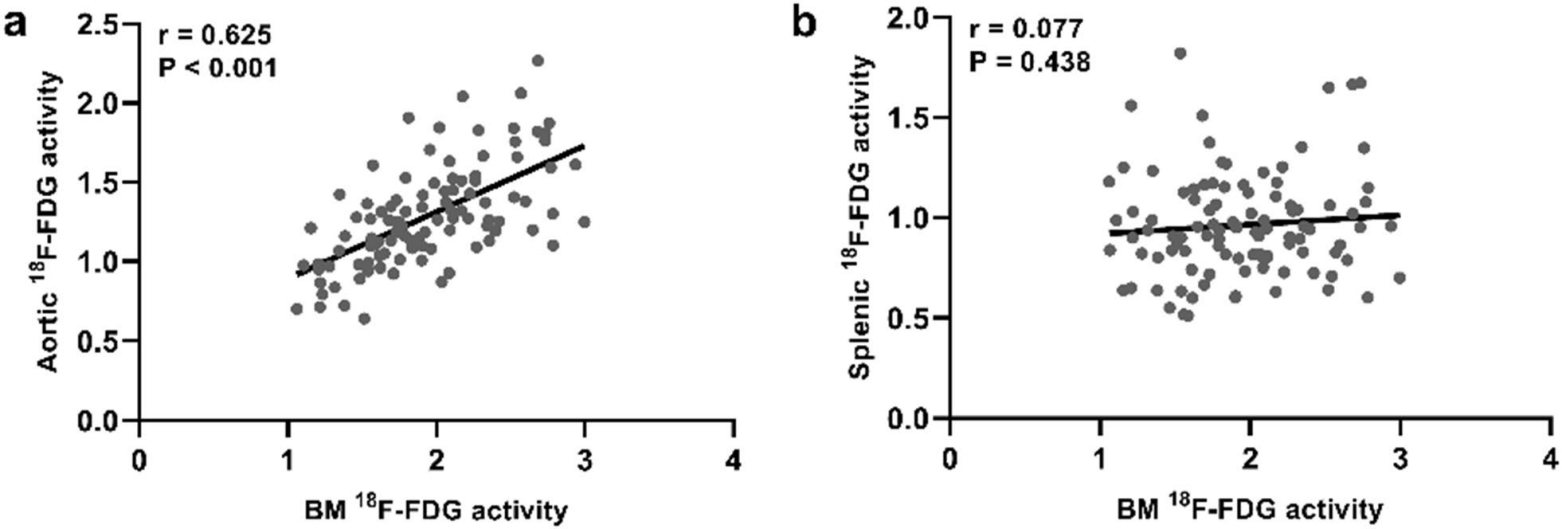
Correlations of BM, aortic and splenic ^18^F-FDG activity. There was a significantly positive correlation between BM and aortic ^18^F-FDG activity (**a**); There was no correlation between BM and splenic ^18^F-FDG activity in the overall study population (**b**). BM, bone marrow

**Fig. 3:**
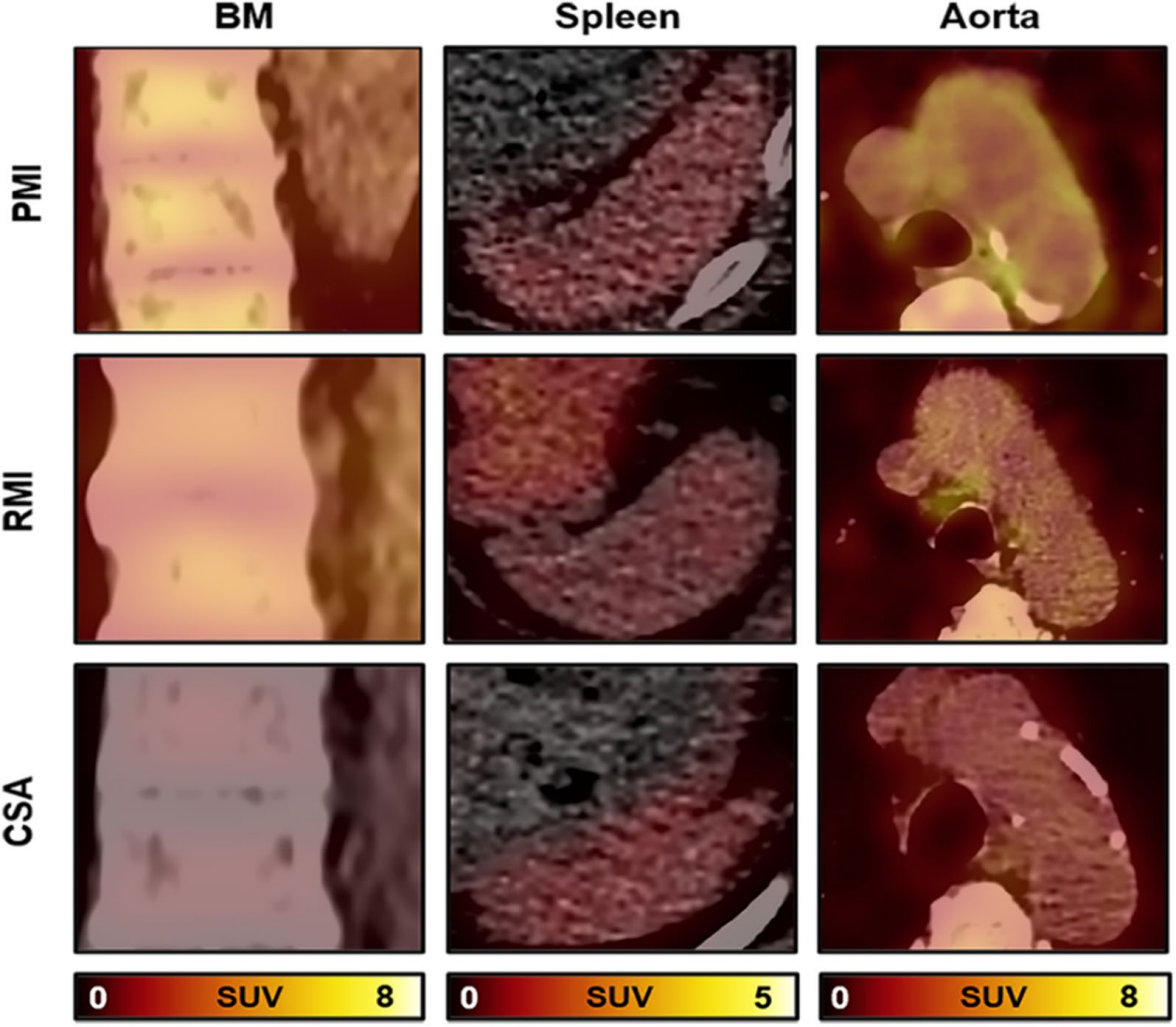
Representative examples of BM, spleen and aorta. BM, Splenic, and aortic ^18^F-FDG activity among the three groups. The coronal fusion of BM, axial fusion of spleen and aorta. BM, bone marrow

TBR_BM_ was highest in the PMI group, intermediate in the RMI group, and lowest in the CSA group [2.26 (0.66) vs. 1.87 (0.45) vs. 1.47 (0.41), P < 0.001] (Fig. 1a). TBR_spleen_ did not differ significantly among the three groups (P > 0.05) (Fig. 1b). TBR_aorta_ in the PMI and RMI groups were significantly higher than that in the CSA group [1.43 (0.43) vs. 1.25 (0.28) vs. 1.04 (0.29), P < 0.001], besides, PMI group had higher TBR_aorta_ than RMI group (Fig. 1c). In addition, TBR_BM_ was significantly correlated with TBR_aorta_ (r = 0.625, P < 0.001) (Fig. 2a), whereas there was no correlation between the TBR_spleen_ and the TBR_aorta_ (Fig. 2b). Representative images of ^18^F-FDG metabolic activity in the BM, spleen, and aorta among the three groups are illustrated in Fig. 3.

### Comparison of TPD, HM, scar, cardiac function, and remodeling among the three groups

There was no significant difference in TPD among the three groups (P > 0.05) (Fig. 1d). In contrast, scar in the RMI group [27.0% (18.0%)] was larger than that in the PMI [18.0% (24.0%)] and CSA [7.5% (21.0%)] groups [RMI group vs. PMI group, P = 0.034; RMI group vs. CSA group, P < 0.001] (Fig. 1e). Correspondingly, HM in the RMI group was smaller than the CSA group [18.0% (15.0%) vs. 28.5% (22.0%), P = 0.024] (Fig. 1f). RMI group [20.0% (10.0%)] exhibited significantly deteriorated LVEF compared to PMI [28.5% (11.0%)] and CSA groups [29.0% (12.0%)] (all P values < 0.001) (Fig. 1g). Additionally, RMI group [EDV, (193.6 ± 68.6) mL; ESV, (155.4 ± 64.8) mL] had more pronounced cardiac remodeling compared with the CSA group [EDV, (129.5 ± 36.0) mL; ESV, (91.9 ± 33.5) mL] (all P values < 0.001, Fig. 1h, i), while EDV did not differ between the PMI and RMI groups [(162.0 ± 65.2) mL vs. (193.6 ± 68.6) mL, P > 0.05] (Fig. 1h). Nevertheless, ESV in the RMI group was greater than PMI group [(155.4 ± 64.8) mL vs. (120.6 ± 59.8) mL, P = 0.032] (Fig. 1i). There was no significant difference in LVEF, EDV, and ESV between the PMI and CSA groups (all P values > 0.05).

### The clinical outcome

**Fig. 4:**
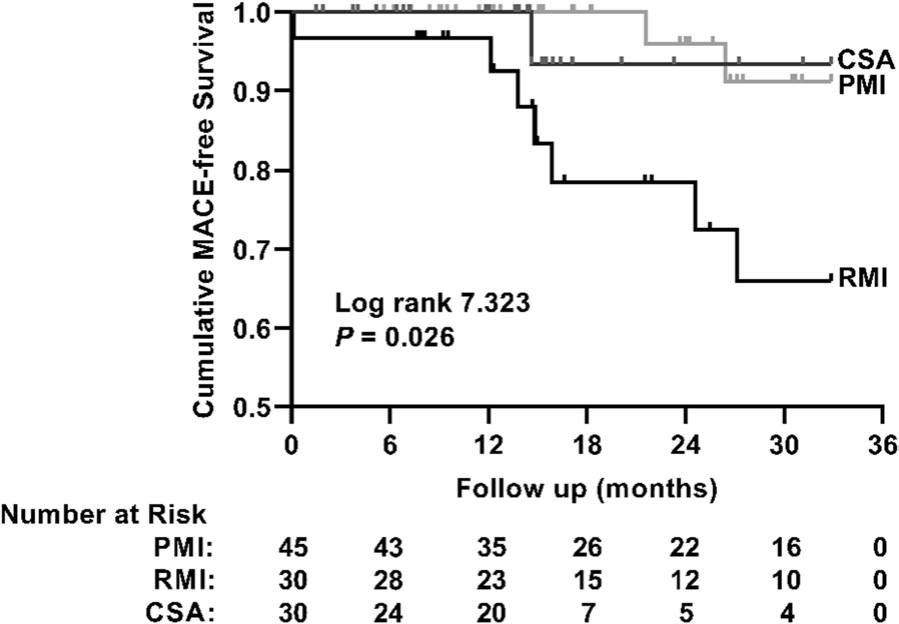
Kaplan–Meier for MACE-free survival curve among the three groups. The cumulative MACE-free survival of patients in the RMI group was significantly lower than that in the PMI and CSA groups. CSA, chronic stable angina; MACE, major adverse cardiac events; PMI, primary myocardial infarction; RMI, recurrent myocardial infarction

During the follow-up period of 16.6 months (interquartile range 11.7–32.0 months), a total of 10 patients suffered from MACE. As shown in Fig. 4, a significant difference of cumulative MACE-free survival was observed among the three groups (P = 0.026). The cumulative MACE-free survival in the RMI group was significantly lower than that in the PMI group [(65.9 ± 10.9) % vs. (91.2 ± 6.0) %, P = 0.014]. No difference of MACE-free survival was observed between RMI and CSA groups, as well as between PMI and CSA groups (all P values > 0.05).

## Discussion

### Main findings

In this study, we evaluated the ^18^F-FDG metabolic activity of BM, spleen, and aorta among PMI, RMI, and CSA patients. We found that the metabolic activity of the BM was significantly decreased in RMI patients in comparison with that in PMI patients. RMI presented a subsequent enlarged myocardial scar, less HM, worsened cardiac dysfunction, and aggravated remodeling as well as poor outcome. Additionally, the ^18^F-FDG metabolic activity in the aorta was significantly associated with the metabolic activity of the BM, which suggested that the inflammatory status of atherosclerosis was influenced by systemic inflammation modulated by the BM.

### RMI patients conferred decreased inflammatory-hematopoietic activation and were associated with an enlarged myocardial scar, less HM, cardiac dysfunction, and aggravated remodeling

The circulating innate immune cells are recruited to the injured myocardium to help myocardial repair after MI [20]. These cells are short-lived, and BM augments leukocyte production to meet the need of organisms [21]. Cremer et al. found that RMI was associated with a state of immune tolerance characterized by reduced emergency hematopoiesis, and reduced accumulation of myeloid cells in the injured mouse heart [7, 22]. However, few studies have demonstrated the metabolic activation of the BM and spleen in humans with RMI [7].

Our results revealed the decreased metabolic activity of hematopoietic tissue (especially BM) in the RMI patients compared with the PMI patients, as assessed by ^18^F-FDG PET. It may indicate the reduction of emergency hematopoiesis after RMI. These findings are consistent with that of Cremer et al. [7]. Further, Dick et al. demonstrated that the depletion of cardiac macrophages impaired cardiac function and worsened myocardial healing post-MI [23]. In our study, RMI patients revealed lower ^18^F-FDG metabolic activity in BM, enlarged myocardial scar, decreased cardiac function, and aggravated remodeling. It may suggest that reduction of macrophage accumulation after RMI presented with detrimental effects on cardiac function and remodeling, while an increased hematopoietic response after PMI could confer superior cardioprotection by the prolonged repairing of the damaged heart tissue and restoring cardiac function.

Hematopoietic stem and progenitor cells progressively differentiate into inflammatory monocytes from the BM to the spleen [24], and inflammatory monocytes subsequently expand to infarcted tissues for enhancing infarct healing [5]. Assmus et al. found that the metabolic activity of BM in MI patients was significantly increased in comparison with chronic heart failure patients [25]. Similarly, we found that the BM activity of MI patients (PMI and RMI) was higher than that in CSA patients. Previous studies have identified early activated splenic ^18^F-FDG activity after MI (mean, 6.3 days; range, 1–10) [26, 27]. Nevertheless, our study found there was no difference in the splenic activity between PMI and RMI patients at the sub-acute phase (30 days) of MI, which may indicate a short half-life of immune response to acute MI. Further studies are needed to determine the half-life of the inflammatory-hematopoietic activation after PMI and RMI in humans and to explore the precise underlying mechanism. Moreover, we found that aortic activity was closely associated with BM activity in the overall patient population. These findings suggested that the inflammatory status of atherosclerosis is influenced by systemic inflammation modulated by the BM [28]. Furthermore, there was a greater aortic inflammatory activity in the PMI patients, compared with the RMI patients, which might indicate prominent monocyte infiltration or longer-term regional immune activation in the vessel after PMI.

### RMI patients were associated with worse outcomes

Innate immunity and cardiac inflammation have been linked to heart failure, which may lead to RMI patients’ low MACE-free survival [29]. Post-MI, the heart recruits millions of circulating myeloid cells to ischemic myocardium. Given the short-lived nature of these cells, BM escalates leukocyte production to compensate for this increased demand. Following an MI, the BM is alerted by signals from the sympathetic nervous system [5] and from circulating mediators produced by the ischemic heart [30]. This process controls hematopoietic stem cells quiescence, proliferation, and differentiation, and subsequently, blood leukocyte levels in response to myocardial injury. Cardiovascular mortality correlates with blood leukocyte levels [31]. Cremer et al. [7] found that white blood cell counts was decreased in patients with RMI compared with that in PMI patients. Correspondingly, in our study, there was a trend that neutrophil was lower in patients with RMI, which might affect recovery by an intricate balance of proinflammatory and tissue repair functions. In theory, reduced emergency hematopoiesis and cardiac recruitment of myeloid cells in a setting of RMI may impair cardiac healing due to insufficient removal of dead cells and altered granulation tissue formation, which results in more necrotic tissue, lack of granulation tissue, and an insufficient collagen matrix [32], and patients may have worse outcomes after RMI. Prospective investigations were warranted to examine hematopoiesis and immune cell levels in patients with RMI, and to further evaluate whether these parameters are related with adverse outcomes.

## Limitations

Our study has several limitations. First, this was a retrospective study with a small sample size, and significant differences among the three groups may exist bias in the cohort, and patients underwent nuclear imaging for the assessment of myocardial viability due to ischemic cardiomyopathy. Second, we did not perform a histopathologic analysis of tissue samples from the BM and spleen. Third, we did not measure the biomarkers of hematopoietic stem cells in BM or monocyte subsets in peripheral blood. Further, there was no difference in the counts of leucocytes and neutrophils in our cohorts, which may be relevant to the subacute phase after MI.

## Conclusions

In this study, we demonstrated that RMI conferred relatively decreased hematopoietic-inflammatory activation in comparison with PMI. Patients with RMI presented subsequent enlarged myocardial scar, worsened cardiac dysfunction, aggravated remodeling, and worse outcomes than that in PMI patients. Further studies of this diminished hematopoiesis and downstream consequences, including the hemopoietic-cardiovascular immune axis in RMI patients, are warranted.

## Data Availability

The data underlying this article will be shared on reasonable request to the corresponding author.

## Abbreviations

BM: Bone marrow
CSA: Chronic stable angina
EDV: End-diastolic volume
ESV: End-systolic volume
FDG: Fluorodeoxyglucose
HM: Hibernating myocardium
LVEF: Left ventricular ejection fraction
MACE: Major adverse cardiac events
MI: Myocardial infarction
MPI: Myocardial perfusion imaging
PET: Positron emission tomography
PMI: Primary myocardial infarction
RMI: Recurrent myocardial infarction
SUV: Standardized uptake value
TBR: Target-to-background ratio
TPD: Total perfusion deficit
